# Supplementary material for: Exploring healthcare providers’ experiences with specialty medication and limited distribution networks
Source: PLoS One. 2022 Aug 15;17(8):e0273040. doi: 10.1371/journal.pone.0273040 (PMC9377589; doi:10.1371/journal.pone.0273040)
Supplement: S2 Table — (DOCX) [file pone.0273040.s002.docx]

**S3 Table. Coding System**

| **Category** | **Label** | **Definition** | **Rules** |
| --- | --- | --- | --- |
| **1** | **Participant description** | **Stakeholder role and experience with LDD--ask about this for formatting** | Designated column - will be generated from linked list |
| 1.1 | Role |  | Designated column - will be generated from linked list |
| 1.1.1 | RN |  | Designated column - will be generated from linked list |
| 1.1.2 | MD |  | Designated column - will be generated from linked list |
| 1.1.3 | NP |  | Designated column - will be generated from linked list |
| 1.1.4 | Other |  | Designated column - will be generated from linked list |
| 1.2 | Clinic |  | Designated column - will be generated from linked list |
| 1.2.1 | Hematology |  | Designated column - will be generated from linked list |
| 1.2.2 | Endocrinology |  | Designated column - will be generated from linked list |
| 1.2.3 | Neurology |  | Designated column - will be generated from linked list |
| 1.2.4 | Peds IBD |  | Designated column - will be generated from linked list |
| 1.2.5 | Peds Rheumatology |  | Designated column - will be generated from linked list |
| 1.3 | Settings of care |  | Code these when you see them |
| 1.3.1 | Inpatient | Identifies inpatient setting | Code these when you see them |
| 1.3.2 | Outpatient | Identifies outpatient setting | Code these when you see them |
| 1.4 | Years of experience with LDD/LDN | Discusses length of experience with LDD/LDN | Code these when you see them |
| 1.5 | Number of pt monthly with specialty meds | Discusses patient volume | Code these when you see them |
| 1.6 | How learned the process | Reflections on how the individual learned to deal with LDD/LDN | Code these when you see them |
| **2** | **Steps involved** | **Steps involved in the process** |  |
| 2.1 | Decision on which med to prescribe | E.g., patient centered care discussion/value-based care |  |
| 2.2 | Communication | Discussion of communication |  |
| 2.2.1 | Internal | Within their facility, provider/nurse/pharmacist |  |
| 2.2.2 | External | E.g., Accredo, Optum/Briova, CVS Specialty, AllianceRX-Walgreens, and Acaria |  |
| 2.2.3 | Patient Communication | Communication directly with patient or caregiver |  |
| 2.3 | Documentation procedures | Talks about how the process is documented |  |
| 2.4 | Timing considerations | E.g., time sensitive, need to start new drug asap |  |
| 2.5 | Teamwork | Delegating tasks, etc. |  |
| 2.6 | Prior authorization/approval | Drug needs prior authorization or approval |  |
| 2.7 | Appeals | Talks about appealing decisions |  |
| 2.8 | Process depends | Identified contingencies that cause variation in the process |  |
| 2.9 | Workaround | Developed solutions to facilitate the process better; (e.g., evidence-based proof to insurance companies) |  |
| 2.10 | Uncertainty | Discusses not knowing or having information |  |
| 2.11 | Changes over time | Reflections on change over time |  |
| 2.12 | Preferences | Specific preferences for how to solve problems |  |
| 2.13 | Other | Other description of steps |  |
| **3** | **Others involved in process** | **Any mention of other people involved in the process** |  |
| 3.1 | MD |  |  |
| 3.2 | Nurse |  |  |
| 3.3 | NP |  |  |
| 3.4 | Pharmacist |  |  |
| 3.5 | Insurance company |  |  |
| 3.6 | Drug representative |  |  |
| 3.7 | Pharmaceutical company |  |  |
| 3.8 | External pharmacy |  |  |
| 3.9 | Patient/caregiver |  |  |
| 3.10 | Other |  |  |
| **4** | **Influences** | **Factors that influence the process** |  |
| 4.1 | Systems factors | Factors associated with the health care systems |  |
| 4.1.1 | Rules/policies/procedures | e.g., has to be written, only one month at a time; insurance company rules, insurance mandates (formulary, step therapy) |  |
| 4.1.2 | Workflow/number of steps | Detailed discussion of workflow and steps involved |  |
| 4.1.3 | Communication/reliance on others | Identifies critical communications |  |
| 4.1.4 | Documentation/administrative steps | How documentation is done |  |
| 4.1.5 | Point of contact | Patient's Point of Contact via health system; contact for external (J? and BB transcripts) |  |
| 4.1.6 | Other | Other Systems factors |  |
| 4.2 | Patient factors | Factors associated with the individual patient |  |
| 4.2.1 | Insurance status | Patient underinsured, having insurance, type of insurance, commercial insurance with copay from manufacturer versus federal insurance (MAP grant) |  |
| 4.2.2 | Compliance | Taking medication on time, stopping medication |  |
| 4.2.3 | Comorbidities | Discusses the role of comorbid conditions |  |
| 4.2.4 | Logistics | Rural, competing priorities, transportation |  |
| 4.2.5 | Other | Other patient factors |  |
| **5** | **Facilitator and barriers** | to get patients the drugs/benefits of specialty pharm |  |
| 5.1 | Barriers/challenges | Barriers/challenges with system/patient/specialty pharmacy |  |
| 5.2 | Facilitars/benefits | Facilitators and benefits of system/patient/specialty pharmacy |  |
| **6** | **Medications** | List of medication discussed in interviews/focus groups; used when discussing medications generally without specific reference to one particular kind |  |
| 6.1 | Gocovri |  |  |
| 6.2 | Xenazine |  |  |
| 6.3 | Austedo |  |  |
| 6.4 | Northera |  |  |
| 6.5 | Nuplazid |  |  |
| 6.6 | Forteo |  |  |
| 6.7 | Strensiq |  |  |
| 6.8 | Kineret |  |  |
| 6.9 | Ilaris |  |  |
| 6.10 | Enbrel |  |  |
| 6.11 | Orencia |  |  |
| 6.12 | Actemra |  |  |
| 6.13 | Other | Used when a specific medication is named but not on the above list |  |
| **7** | **Suggestions** |  |  |
| 7.1 | Practice/Healthcare-specific suggestions | Healthcare structures and processes |  |
| 7.2 | Economic | Economic/cost changes |  |
| 7.3 | Other | Other suggestions |  |
