# Supplementary material for: Exploring healthcare providers’ experiences with specialty medication and limited distribution networks
Source: PLoS One. 2022 Aug 15;17(8):e0273040. doi: 10.1371/journal.pone.0273040 (PMC9377589; doi:10.1371/journal.pone.0273040)
Supplement: S1 Text — (DOCX) [file pone.0273040.s004.docx]

**S2 Text: Semi-Structured Interview/Focus Group Guide**

[Introduction, overview of study purpose, informed consent, audio recording]

LDD: Drugs that are required to be dispensed by a few, select pharmacies. For the purposes of this interview, we will discuss those medications that are not able to be dispensed by Vanderbilt Specialty Pharmacy. For your clinic, this includes….

1. What is your role in patient care (*prescriber, medication access coordinator, other)?*
2. Give us an overview of your experience with LDDs—how many have you dealt with? Which ones? How often do you prescribe/deal with LDDs?

**Domain 1: Workflow**

1. How are you informed/educated that a medication is subject to LDN?
   1. I am not
   2. Drug manufacturer
   3. Pharmacist
   4. PBM
   5. Specialty Pharmacy leadership
2. Once you find out a drug has to be dispensed through an LDN, what is your process?
3. How does this process compare to medications that are not LDN?
   1. Time requirements
   2. Documentation requirements (*PA, appeal, ongoing documentation requirements, REMS requirements*)
   3. Communication requirements or process variance (*physician, patient, pharmacy*)

**Domain 2: Patient Outcomes**

1. How do you think patients are affected by a medication being required to be dispensed through an IDN
   1. Time to treatment
   2. Time to approval
   3. Final access to the medication (i.e. do they get it)
   4. Financial
   5. Disease progression
2. Can you give us an example of a patient being affected by LDN

**Domain 3: Clinical Practice** (Prescribers only)

1. How often do you prescribe LDN medications? (compared to non-LDDs)
2. How does your frequency of prescribing impacted by a product being LDN (*prescribe less, does not affect, etc.)?*
3. How likely are you to prescribe a therapeutic alternative that is not subject to LDN in lieu of LDN drug

**Other:**

- Do you have any additional comments regarding the impact of LDNs on patients or providers from your perspective?
