# Supplementary material for: Exploring healthcare providers’ experiences with specialty medication and limited distribution networks
Source: PLoS One. 2022 Aug 15;17(8):e0273040. doi: 10.1371/journal.pone.0273040 (PMC9377589; doi:10.1371/journal.pone.0273040)
Supplement: Attachment — Submitted filename: R2R.docx [file pone.0273040.s006.docx]

| **Response to Reviewers** | |
| --- | --- |
| **Reviewer Comment** | **Authors Response** |
| **Editor’s Notes** | |
| Please ensure that your manuscript meets PLOS ONE's style requirements, including those for file naming. The PLOS ONE style templates can be found at | *We appreciate this reminder and have reviewed our manuscript to ensure it meets styling requirements. The template was appreciated.*  *File naming updated:*  *S1 Table 1. ISSM COREQ Checklist*  *S2 Text. Semi-Structured Interview.Focus Group Guide*  *S3 Table 2. Coding System (updated number to reflect new supplementary material provided)*  *File added with same naming format:*  *S4 Table 3. Coded Minimal Data Set* |
| In the Methods section of the manuscript, please consider including more information on the number of interviewers, their training and characteristics; and please provide the interview guide used. | *We have modified the interviewer description to be more detailed and clear (page 5, lines 108-112).*  *We have uploaded the semi-structured interview and focus group guide (S2 Text. Semi-Structured Interview.Focus Group Guide).* |
| In your Data Availability statement, you have not specified where the minimal data set underlying the results described in your manuscript can be found. PLOS defines a study's minimal data set as the underlying data used to reach the conclusions drawn in the manuscript and any additional data required to replicate the reported study findings in their entirety. All PLOS journals require that the minimal data set be made fully available. For more information about our data policy, please see <http://journals.plos.org/plosone/s/data-availability>. | *We have uploaded a minimal data set that was used for coding and analysis. Please let us know if this is not acceptable.* |
| Please upload a copy of Supporting Information Table 2 which you refer to in your text on page 5. | *I confirmed that this information was uploaded in the original submission. It is now labeled S1 Table 1. ISSM COREQ Checklist. Based on the journal guidelines, we thought this name was acceptable, but we did remove the Complete and the underscores please let us know if there is a preference for a different name. Guideline excerpt:*  Authors may use almost any description as the item name for a supporting information file as long as it contains an “S” and number. For example, “S1 Appendix” and “S2 Appendix,” “S1 Table” and “S2 Table,” and so forth. |
| Please review your reference list to ensure that it is complete and correct. If you have cited papers that have been retracted, please include the rationale for doing so in the manuscript text, or remove these references and replace them with relevant current references. | *We have reviewed the reference list and confirm they are complete and correct. We updated reference #26 as this data has now been published* |
| **Reviewer #1** | |
| We have reviewed the study reporting perspectives from specialty providers and clinic staff on the impact of limited distribution drug networks in real-world practice. The authors have shown that participants noted that IHSSPs reduce clinic burden by helping patients access, afford, and remain on therapy. When IHSSPs are included in a drug’s LDN, healthcare providers report high satisfaction, more confidence in prescribing specialty medications, improved workflow efficiency, and better patient outcomes. Manufacturers who wish to distribute specialty medication through LDNs should create and communicate transparent criteria for specialty pharmacies to meet distribution goals. We find the study contributing to the knowledge in service delivery in healthcare. We have included the MS highlighting areas we think may need to be corrected or included. Key words and abbreviations may be included in the MS as well. | *We appreciate the reviewer’s comments and feedback on the manuscript. Suggested grammatical edits in the attached document that was provided have been incorporated in the updated manuscript. We have carefully reviewed the manuscript and added additional grammatical changes where appropriate.* |
