# Supplementary material for: Exploring healthcare providers’ experiences with specialty medication and limited distribution networks
Source: PLoS One. 2022 Aug 15;17(8):e0273040. doi: 10.1371/journal.pone.0273040 (PMC9377589; doi:10.1371/journal.pone.0273040)
Supplement: Attachment — Submitted filename: Response to Reviewers_2.docx [file pone.0273040.s007.docx]

Autumn Zuckerman, PharmD, BCPS, AAHIVP, CSP

726 Melrose Ave

Nashville, TN 37211

July 14, 2022

Dear Dr. Bianchi and colleagues,

Thank you for the opportunity to submit another revised version of our manuscript, “Exploring healthcare providers' experiences with specialty medication and Limited Distribution Networks.” Contained within this letter are detailed responses to each reviewer’s concerns, comments, and requested changes.

Please note, we have retained the track changes previously added on the last peer review. Please do let us know if there are any additional items to be addressed. Thank you!

Sincerely,

Autumn Zuckerman, PharmD, BCPS, AAHIVP, CSP

Corresponding Author

| **Response to Reviewers** | |
| --- | --- |
| **Reviewer Comment** | **Authors Response** |
| **Reviewer #1** | |
| We have made comments to the Authors in our initial review where we had accepted the MS for publication. | *We appreciate the reviewer’s comments and previous feedback on the manuscript.* |
| **Reviewer #2** | |
| Thank you for the opportunity to review this manuscript. Overall, the manuscript reads well and is easy to follow. | *Thank you for these kind words and feedback.* |
| It is unclear how the authors ensured data saturation, which is an important process for deciding how many people to interview in qualitative studies | *We appreciate you identifying this omission. We’ve added to page 6, line 130 that data saturation was not assessed. Because there was a relatively small sample of providers/clinics able to be interviewed, we chose a priori to perform interviews/focus groups on all potential participants regardless of data saturation.* |
| It appears that the interviewee selections are mainly based on convenience rather than purposive sampling. Ten out of 14 participants (71.5%) are nurses/nurse practitioners. As such, the findings mostly represent nurses’ perspectives rather than the broader healthcare providers perspectives. | *The sampling was purposive as we used criteria that the clinic had to be familiar with and utilize LDDs in practice. The sample has a larger amount of nurses because they are typically the staff that address access issues for LDDs and therefore more knowledgeable on the topic. We’ve added to the limitations, page 28, line 586 that most of the participants were nurses, which contributes to limited generalizability.* |
| Although clinical pharmacists are the main players/stakeholders in LDN/VSP, the authors failed to incorporate pharmacists’ perspectives in the study, which is a missed opportunity to better understand the complexity of the IHSSP and LDN systems. Also, it would have been better to incorporate manufacturers’ perspectives. Unless we hear all stakeholders’ views, it would be difficult to come up with workable solutions. | *We appreciate this perspective. The focus of this paper was to elucidate provider and clinic staff experience with LDNs rather than giving a sweeping view of the current LDN landscape. There is much more research to be done in this area as LDNs continue to increase and gathering opinions from pharmacists and manufacturers would be a great next step. We’ve added this to future directions (page 28, line 581).* |
| It is unclear why the authors didn’t incorporate the field notes in their data analysis. | *We did not keep field notes for this study. Interviews were recorded and transcribed, providing the primary qualitative data. This is noted on page 9, line 155. We believe that the data provided meets PLOS One requirements of a minimal data set.* |
| “Member check” is an important step in ensuring the trustworthiness/quality of qualitative data. However, the authors didn’t provide an opportunity for the interview to comment on the findings or edit/confirm the interview transcripts. | *Thank you for this feedback. We have added this as a limitation to our methods (page 29, line 596). The experience of the Vanderbilt Qualitative Research Core is that providers are busy clinicians with minimal time and usually lack of desire to further review transcripts. However, we did not note this in the manuscript as this anecdotal experience may vary.* |
| The focus group discussions only included two or three people. It is usually not recommended to have FGDs with less than five or six participants. | *This is a great point. We were unable to have larger focus groups due to limited staff with the knowledge/experience needed to speak to the topic. Additionally, we wanted to keep the focus groups specific to each clinic so that we could understand if certain clinical areas are affected differently than others, further limiting our potential to combine areas and enlarge our focus group size. We’ve added this as a limitation (page 29, line 591)* |
| The authors should also discuss the pros and cons of a non-health professional interviewer. Although having a non-health professional as an interviewer may help to avoid bias, there are several limitations of this approach in health research, and this should be properly discussed in the discussion section. | *We agree with the reviewer that this should be addressed and have added a discussion on pros/cons of this approach to the discussion (page 29, line 599)* |
| Line 576-578: “Participants were individually selected by investigators based on their experience with prescribing or coordinating care for specialty medications that are distributed through an LDN; thus, sampling bias may have occurred.” I don’t think this is relevant for a qualitative study. In qualitative studies, bias cannot be avoided, they rather should be acknowledged. The concept of bias in qualitative and quantitative research is also different. | *Thank you for this feedback. We have updated the limitations to soften the language that that this recruitment method may have introduced limitation and changed verbiage to selection bias (page 29, line 595).* |
| Don’t mix methods and results. Some of the methods section contain results, for example, lines 133 to 141 (including table 1). | *We have moved the participant information to results as requested.* |
| What is the purpose of reporting the ethnicity/race of participants as well as their age and sex in Table 1? None of these characteristics was used to inform the data analysis. Perhaps, looking at the ethnicity/race of the participants, it appears that only practitioners from one ethnic group are recruited. Again, this is another example of missed opportunity by the researchers to include diverse perspectives! | *We have retained providing the race/ethnicity, age, and sex to give reader’s insight into the makeup of participants as we believe this is typical in most qualitative research studies. As previously noted, we were limited in our ability to include diverse perspectives based on the small number of healthcare practitioners with robust experience with LDNs at our institution. We certainly would have liked to have a more diverse sample to engage. We’ve added the limited diversity in our sample to limitations (page 28, line 586)* |
| Please revise the limitation section of the discussion according to the above comments and suggestions. | *We have revised limitations as noted above.* |
